# Quantitative Detection of Pericardial Adhesions Using Four-Dimensional Computed Tomography: A Novel Motion-Based Analysis Framework

**DOI:** 10.3390/bioengineering12030224

**Published:** 2025-02-22

**Authors:** Tong Ren, Shuo Wang, Nan Cheng, Zekun Feng, Menglu Li, Li Zhang, Rong Wang

**Affiliations:** 1 Department of Adult Cardiac Surgery, Senior Department of Cardiology, The Six Medical Center of PLA General Hospital, Fucheng Road, Haidian District, Beijing 100048, China; rentong8713@163.com (T.R.); cn86919@163.com (N.C.); fengzk1990@gmail.com (Z.F.); 2 Chinese PLA Medical School, Fuxing Road, Haidian District, Beijing 100089, China; 3 Key Laboratory of Particle and Radiation Imaging, Department of Engineering Physics, Ministry of Education, Tsinghua University, Beijing 100084, China; shuo-wan19@mails.tsinghua.edu.cn; 4 Department of Diagnostic Radiology, The Six Medical Center of PLA General Hospital, Fucheng Road, Haidian District, Beijing 100048, China; 15801150136m@sina.cn

**Keywords:** pericardial adhesions, 4D CT, epicardial adipose tissue, cardiac surgery, motion quantification

## Abstract

Objective: Pericardial adhesions can unexpectedly occur prior to cardiac surgery or catheter ablation, even in patients without known risk factors, potentially increasing procedural risks. This study proposed and validated a novel, quantitative, and noninvasive method for detecting pericardial adhesions using four-dimensional computed tomography (4D CT). Methods: We evaluated preoperative 4D CT datasets from 20 patients undergoing cardiac surgery with and without pericardial adhesions. Our novel approach integrates expert-guided pericardial segmentation, symmetric diffeomorphic registration, and motion disparity analysis. The method quantifies tissue motion differences by computing the displacement fields between the pericardium and epicardial adipose tissue (EAT), with a particular focus on the left anterior descending (LAD) region. Results: Statistical analysis revealed significant differences between adhesion and non-adhesion groups (*p* < 0.01) using two newly developed metrics: peak ratio (PR) and distribution width index (DWI). Adhesion cases demonstrated characteristic high PR values (>100) with low DWI values (<0.3), while non-adhesion cases showed moderate PR values (<50) with higher DWI values (>0.4). Conclusions: This proof-of-concept study validated a novel quantitative framework for assessing pericardial adhesions using 4D CT imaging and provides an objective and computationally efficient tool for preoperative assessment in clinical settings. These findings suggest the potential clinical utility of this framework in surgical planning and risk assessment.

## 1. Introduction

The pericardium is a double-layered membranous structure surrounding the heart, comprising two avascular layers, the parietal pericardium and the visceral pericardium (epicardium), with a potential space—the pericardial cavity—between these two layers. The pericardial cavity, containing serous fluid, minimizes friction during cardiac motion. It anchors the heart within the thoracic cavity, restricts excessive dilation, and provides mechanical protection. The pericardium also serves as an immunological barrier as well as maintains optimal cardiac geometry and function. These properties ensure smooth cardiac dynamics and support physiological homeostasis while preventing external damage or pathological overexpansion [1]. Pericardial adhesions are defined as the formation of fibrous connections between the pericardial layers resulting from inflammation, fibrosis, or other pathological changes within the pericardial cavity. These adhesions can restrict the heart’s movement and surrounding structures, leading to the blurring of anatomical layers [2]. This pathology may increase the risk and complexity of cardiac surgery by hindering intraoperative exposure and compromising the surgeon’s ability to visualize the operative field clearly [3]; unanticipated pericardial adhesions encountered during minimally invasive cardiac surgery may compel the surgeon to modify the planned approach, potentially converting it to a more invasive or open procedure, thereby increasing surgical trauma and risk. Additionally, during epicardial catheter ablation, pericardial adhesions can limit catheter maneuverability within the epicardial region, reducing the precision of ablation and potentially prolonging the procedure; this may decrease the short-term success of the intervention [4]. The primary causes of pericardial adhesions include a history of pericarditis, prior cardiac surgery, and radiation therapy [5]. However, adhesions have also been observed in patients without these risk factors, with an incidence ranging from 8% to 14.7% [6], highlighting the importance of preoperative evaluation.

Slippage between the visceral and parietal pericardium is observed in healthy individuals, while the absence of this slippage characterizes those with anatomical pericardial adhesions [7]. Prior investigations have utilized two-dimensional (2D) echocardiography and quantitative tissue Doppler imaging to assess pericardial adhesions through the analysis of the dynamic relationship between pericardial and myocardial motion [8]; cardiac magnetic resonance imaging (CMR), renowned for its exceptional tissue characterization capabilities, has been utilized in investigative studies to evaluate pericardial adhesions through the comprehensive analysis of tissue compositional properties—including inflammatory changes, calcification patterns, and protein content distribution—in combination with the assessment of relative cardiac motion restriction [9]; meanwhile, recent studies have reported favorable outcomes in the evaluation of preoperative pericardial adhesions using the invasive epicardial carbon dioxide insufflation (EpiCO2) technique [10]. However, echocardiography is limited by the need for an adequate imaging window and its inability to characterize tissues fully. While providing detailed images, CMR has limitations, including longer acquisition times, higher costs, and potential interference with intracardiac metal implants [11]. While EpiCO2 represents a reliable diagnostic method, it requires intraoperative implementation and is considered an invasive procedure, thereby incurring additional surgical costs, extending operative time, and introducing additional procedural risks. Computed tomography (CT) is particularly effective in visualizing cardiac structures, pericardial thickening, and calcification, as well as generating cardiac motion sequences. With four-dimensional computed tomography (4D CT) [12], which maintains the same high spatial resolution and excellent tissue contrast as conventional CT imaging, dynamic cardiac motion sequences can now be generated while preserving detailed anatomical information. While 4D CT presents certain challenges compared to conventional contrast-enhanced CT, such as extended acquisition time (approximately 20 min), increased institutional data storage requirements, and prolonged image postprocessing duration, it nevertheless remains the most accessible, efficient, and cost-effective modality for acquiring cardiac motion and three-dimensional anatomical information. However, there is a lack of methods for quantitatively dynamic analyzing pericardial adhesions using CT.

Epicardial adipose tissue (EAT) is defined as fat located between the myocardium and the visceral pericardium, in direct contact with the pericardium. It is categorized into pericoronary EAT (surrounding the coronary arteries) and myocardial EAT (overlying the myocardium). EAT covers approximately 80% of the heart’s surface and can be quantitatively measured using cardiac imaging techniques [13]. Given the close anatomical relationship between EAT and the pericardium, this study aimed to develop a novel, noninvasive, and quantifiable method for assessing pericardial adhesions by analyzing the relative motion between EAT and the pericardium using preoperative CT datasets, with preliminary validation of the method’s feasibility.

## 2. Materials and Methods

### 2.1. Participants and Imaging

This study included 20 patients who underwent multiphase reconstructive computed tomography angiography (CTA) before surgery. Of these, 13 patients were undergoing their first cardiac surgery, with a mean age of 53.2 years, and 12 were men. The remaining seven patients were undergoing redo cardiac surgery (with autologous pericardial suturing during the first procedure), with a mean age of 55.5 years, six of whom were men. The patient demographics are summarized in Table 1.

Operative reports confirmed the presence of extensive pericardial adhesions in all patients undergoing redo surgery and the absence of adhesions in all patients undergoing first-time surgery. Data processors were blinded to the pericardial adhesion status of the patients throughout this study. The Ethics Committee of the General Hospital of the Chinese People’s Liberation Army accepted this protocol (S2024-580), and written informed consent was obtained from all participants.

CT images were obtained using a GE APEX CT scanner (Revolution Apex, GE Healthcare, Milwaukee, MI, USA) in sinus rhythm and during end-expiratory breath-hold. The images were reconstructed in 11 temporal phases, with each assigned an R-R value ranging from −5 to 106%. The in-plane resolution was 0.23 × 0.23 mm, and the slice thickness was 0.625 mm in increments of 0.625 mm. The mean effective dose determined using the European criteria for multilayer CT was 10.49 mSv.

### 2.2. Pericardial EAT Motion Analysis Methodology

**Figure 1:**
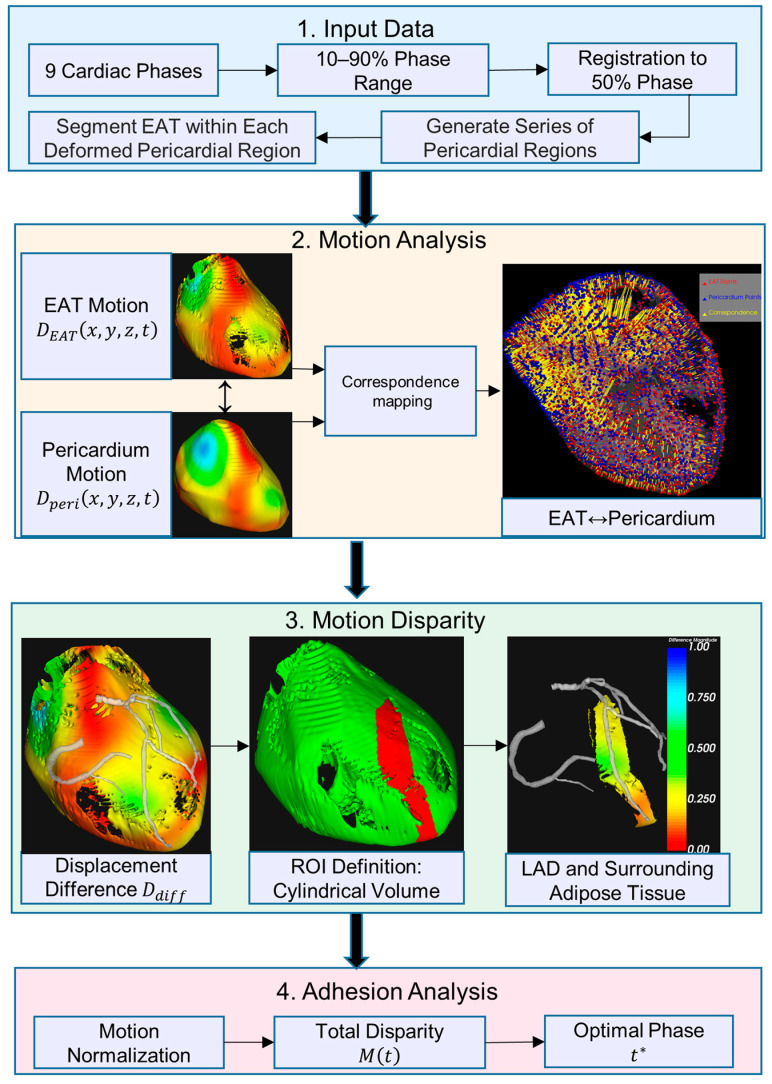
Overview of the cardiac motion analysis pipeline for EAT and pericardium. The heatmap utilizes color gradients to visualize the motion characteristics of the cardiac surface. As illustrated in the color spectrum of ‘3, motion disparity’, red regions correspond to areas of minimal motion amplitude, whereas blue regions indicate zones of pronounced, substantial movement. $t^{*}$ represents the cardiac phase at which the cumulative motion difference between the pericardium and EAT reaches its peak value.

The workflow consists of four main stages, as shown in Figure 1: (1) input data: cardiac CT images from nine phases (10–90%) are registered to the 50% reference phase, where transformations are applied to the reference pericardial region, followed by cardiac cycle sequence generation and EAT segmentation in deformed regions; (2) motion analysis: generation of 3D displacement fields for both pericardium and EAT motion tracking; (3) motion disparity: calculation of differential motion within a cylindrical ROI around the left anterior descending (LAD); and (4) adhesion analysis: normalization of motion parameters and determination of the optimal cardiac phase with maximum motion disparity. Colored blocks represent different processing stages: input (blue), motion processing (orange), disparity analysis (green), and final output (red).

#### 2.2.1. Pericardial and EAT Segmentation Pipeline

The pericardium was segmented using 3D-Slicer v5.2.2 (Brigham and Women’s Hospital and Massachusetts Institute of Technology, Boston, MA, USA) by an expert with over five years of experience in CTA image interpretation. The reference phase, selected during the end-diastolic phase when myocardial motion is minimal, provided optimal image quality for pericardial boundary delineation. A systematic slice-by-slice segmentation protocol was implemented, with the region of interest (ROI) defined between the diaphragm (inferior boundary) and the pulmonary artery bifurcations (superior boundary). The segmentation workflow was initiated in the axial plane, where the expert initially delineated the pericardial contours on the central slice of the image volume, subsequently proceeding in three-slice intervals in both superior and inferior directions. Coronal and sagittal reconstructions were utilized as reference planes to enhance segmentation accuracy. In regions with ambiguous pericardial boundaries, the expert employed interpolation based on adjacent annotated slices and anatomical knowledge to ensure precise delineation. Following manual contour delineation, automated image interpolation was applied to complete the intermediate unannotated slices, followed by median filtering for contour smoothing. A 3D annotated label map was generated to facilitate comprehensive expert review and refinement of any potential segmentation inconsistencies.

Subsequently, nine cardiac phases (10–90%) were registered to the reference 50% phase using a symmetric diffeomorphic algorithm [14]. The registration employed the symmetric normalization (SyN) approach [15], which generates diffeomorphic mappings $\varphi \left(x,t\right):\Omega \times t\;\;\Omega$ that are both differentiable and invertible, obtained by integrating a time-dependent velocity field $v\left(x,t\right)$. The transformation is governed by $\frac{d\varphi \left(x,t\right)}{dt}=v\left(\varphi \left(x,t\right),t\right)$. To achieve symmetry, the transformation is decomposed into two components $\varphi_{1}$ and $\varphi_{2}$, optimizing the following variational energy:

(1) $$E\left(v\right)=\int_{0}^{0.5}\prod \left(I,J\circ \varphi_{1}\right)dt+\int_{0.5}^{1}\prod \left(J,I\circ \varphi_{2}\right)dt+\lambda \int_{0}^{1}{\left\|v\left(x,t\right)\right\|}_{L}^{2}dt$$

where ∏ denotes the image similarity metric, λ controls the regularization strength, and I and J represent the fixed and moving images, respectively. The composition $J\circ \varphi_{1}$ indicates that the spatial coordinates are first mapped to new positions through the transformation $\varphi_{1}$, followed by sampling the image J at these transformed coordinates to obtain the corresponding image intensity values.

This registration process generated a sequence of transformations represented by a time-varying 3D displacement field. The pericardial region at the reference phase, denoted as $\Omega_{peri}^{ref}$, was deformed according to the sequence of transformations to generate a complete cardiac-cycle series of pericardial regions. Specifically, let $\phi_{t}:R^{3}⟶R^{3}$ denote the transformation at cardiac phase t, where $t\in \left\{10\%,20\%,\ldots ,90\%\right\}$. The deformed pericardial region at each phase t can be expressed as $\Omega_{peri}^{t}=\phi_{t}(\Omega_{peri}^{ref})$.

**Figure 2:**
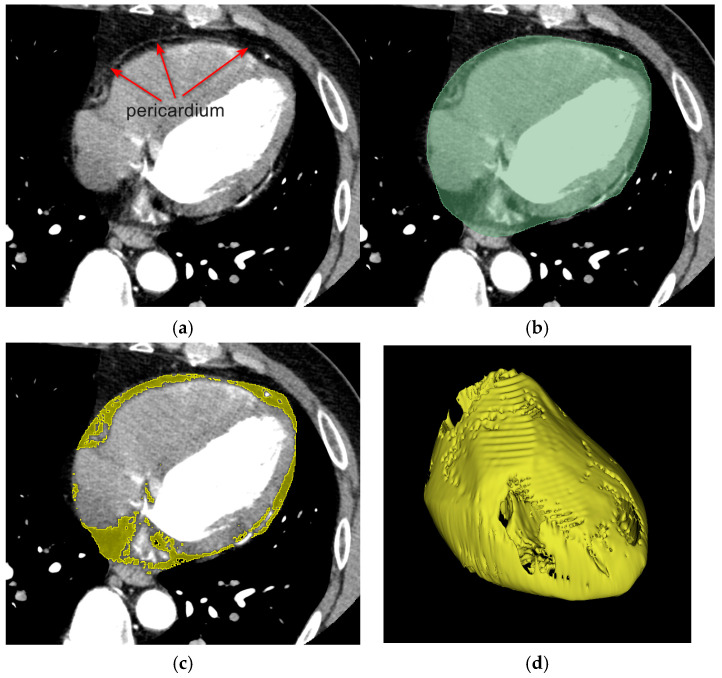
EAT segmentation workflow. (**a**) Coronary CTA image showing pericardium as a thin white line. (**b**) Manual segmentation of pericardial region (green). (**c**) Threshold-based segmentation of adipose tissue within pericardial mask (yellow). (**d**) Reconstructed 3D surfaces of EAT.

Each transformed region inherits the spatial resolution and topological properties of $\Omega_{peri}^{ref}$, while capturing the phase-specific anatomical deformation. Within each deformed pericardial region $\Omega_{peri}^{t}$, the EAT, denoted as $\Omega_{EAT}^{t}$, was segmented using a threshold-based approach. Specifically, contiguous voxels with Hounsfield units ranging from −190 to −30 were identified and classified as EAT [16]. Based on the established radiodensity characteristics of adipose tissue, this segmentation method enables precise quantification of the EAT distribution across all cardiac phases, as depicted in Figure 2.

#### 2.2.2. Motion Field Computation and Point Correspondence

Initially, the displacement fields of the dynamic pericardium and EAT were calculated separately for each cardiac phase. The displacement vectors, denoted as $D_{peri}\left(x,y,z,t\right)\in R^{3}$ and $D_{EAT}\left(x,y,z,t\right)\in R^{3}$ for the pericardium and EAT, respectively, were computed at each spatial position $\left(x,y,z\right)\in \Omega$ and temporal phase $t\in \left[0,1\right]$, where Ω represents the spatial domain. For any given normalized time point t, the displacement vector $D_{peri}\left(x,y,z,t\right)$ was defined as

(2) $$D_{peri}\left(x,y,z,t\right)=\left\{\begin{array}{c} P\left(x,y,z,0\right)-P\left(x,y,z,1\right),\begin{array}{cc} & \;\mathrm{if}\; \end{array}t=0\\ P\left(x,y,z,t\right)-P\left(x,y,z,t-∆t\right),\begin{array}{cc} & \mathrm{if}\;t>0 \end{array} \end{array}\right.$$

where $P\left(x,y,z,t\right)$ represents the spatial coordinates of the pericardium points at the normalized phase t, and ∆t represents the normalized time interval between consecutive frames. Similarly, the displacement vector $D_{EAT}\left(x,y,z,t\right)$ for the EAT was calculated following the same formulation, with $P\left(x,y,z,t\right)$ representing the spatial coordinates of the EAT points. To ensure cyclic cardiac motion, the displacement at t=0 was calculated using the first and last phases, creating a cyclic representation of cardiac motion. For each phase, we established the point correspondences between the EAT and pericardium by implementing a nearest-point mapping. Specifically, for any point $p\in \Omega_{EAT}^{t}$, its corresponding point on the pericardium $q\in \Omega_{peri}^{t}$ was determined by minimizing the Euclidean distance:

(3) $$q=\arg\min\left\{{\left\|p-s\right\|}_{2}:s\in \Omega_{peri}^{t}\right\}$$

where ${\left\|\cdot \right\|}_{2}$ denotes the Euclidean norm, $\Omega_{EAT}^{t}$ represents the spatial domain of the EAT, and $\Omega_{peri}^{t}$ represents the spatial domain of the pericardium.

#### 2.2.3. Motion Disparity Analysis and ROI Definition

The motion difference between corresponding points was quantified by computing the displacement vector difference between each EAT point and its nearest pericardial point. For each corresponding point pair $\left(p,q\right)$, the relative displacement difference $D_{diff}$ was calculated as

(4) $$D_{diff}\left(p,q,t\right)=D_{EAT}\left(p,t\right)-D_{peri}\left(q,t\right)$$

where $D_{diff}\left(p,q,t\right)\in R^{3}$ represents the local motion disparity at time t. The magnitude of this difference ${\left\|D_{diff}\left(p,q,t\right)\right\|}_{2}$ provides a scalar measure of the motion inconsistency between the pericardium and EAT, with larger values indicating greater motion disparity between the two tissues.

The quantitative analysis of pericardial adhesions in the region adjacent to the coronary arteries required a standardized selection of the assessment range to ensure consistency across all 20 datasets while maintaining reproducibility and anatomical accuracy. The main branches of the coronary arterial system include three primary vessels. The LAD and left circumflex (LCX) artery both arise from the left main coronary artery, while the right coronary artery (RCA) originates directly from the ascending aorta. These three major vessels, along with their subsequent branches, form the essential vascular network that supplies oxygenated blood to the cardiac muscle. Due to the complexity of coronary lesions in patients undergoing coronary artery bypass grafting (CABG), some patients had occlusion of the RCA, which was not visible on CTA images. Additionally, some patients with a history of secondary surgery had a metallic prosthetic mechanical valve implanted in the mitral position, resulting in the absence of the visualization of the LCX. The LAD is considered the most critical vessel in the coronary system due to its extensive functional and surgical significance. It supplies approximately 40–50% of the myocardium, including the anterior wall, anterolateral wall, apex of the left ventricle, and anterior two-thirds of the interventricular septum. LAD occlusion can result in severe anterior myocardial infarction, which has high mortality rates, making its revascularization, particularly using the left internal mammary artery, a fundamental and crucial step in CABG procedures, which provides the best long-term patency outcomes. The LAD traverses the anterior interventricular groove, where pericoronary adhesions frequently occur, while the proximal LAD, deeply embedded in the EAT, is relatively protected from injury and potentially influenced by aortic motion; we focused our quantitative assessment on the middle and distal segments of the LAD. This ROI selection ensured more reliable motion analysis and better reflected the interaction between the coronary vessel and surrounding tissues. The ROI was extracted from $\Omega_{EAT}^{t}$ along the middle and distal segments of the LAD. Specifically, we defined a cylindrical assessment volume centered on the LAD centerline, formulated as

(5) $$\Omega_{ROI}^{t}=\left\{p\in \Omega_{EAT}^{t}:{\left\|p-C\left(s\right)\right\|}_{2}\leq r,s\in [s_{mid},s_{dist}]\right\}$$

where $C\left(s\right)$ represents the LAD centerline parameterized by arc length s; $s_{mid}$ and $s_{dist}$ denote the starting points of middle and distal segments, respectively; and r=6 mm is the cylinder radius. $\Omega_{ROI}^{t}$ encompasses all epicardial adipose tissue points within the cylindrical volume, ensuring comprehensive coverage of the pericoronary region while maintaining anatomical relevance.

#### 2.2.4. Motion Disparity Quantification and Normalization

For each patient, we first identified the maximum motion difference magnitude across all cardiac phases and point pairs:

(6) $$D_{max}=\underset{p\in \Omega_{\mathrm{EAT}}^{t}}{\max}{\left\|D_{diff}\left(p,q,t\right)\right\|}_{2}$$

The motion difference magnitudes were then normalized to the range [0, 1]:

(7) $${\hat{D}}_{diff}\left(p,q,t\right)=\frac{{\left\|D_{diff}\left(p,q,t\right)\right\|}_{2}}{D_{max}}$$

Subsequently, the total motion disparity $M\left(t\right)$ at each cardiac phase t was computed by integrating the normalized displacement differences across all corresponding EA–pericardium point pairs within the defined ROI:

(8) $$M\left(t\right)=\sum_{p\in \Omega_{ROI}^{t}}{\left\|{\hat{D}}_{diff}\left(p,q,t\right)\right\|}_{2}$$

This normalization step ensures standardized comparison across different patients and cardiac phases while preserving the relative motion patterns between the EAT and pericardium. The optimal phase $t^{*}$ exhibiting the maximum motion disparity was then determined by

(9) $$t^{*}=\arg\max\left\{M\left(t\right)\right\}$$

where $t^{*}$ represents the cardiac phase at which the cumulative motion difference between the pericardium and EAT reaches its peak value. This phase-specific analysis enables the identification of the temporal instance where the motion inconsistency between the two tissues is most pronounced, providing a crucial time point for subsequent clinical assessment and visualization.

## 3. Results

Firstly, we validated the reliability of our motion analysis and its conformity with standard cardiac motion patterns, which was essential for ensuring accurate results; early systole was identified as the phase with the greatest difference in motion. Next, we conducted a quantitative analysis comparing cases with and without pericardial adhesions; our proposed metrics effectively quantify the pericoronary motion differences and establish their reliability for adhesion pattern analysis.

### 3.1. Reliability Validation of Cardiac Motion Analysis

**Figure 3:**
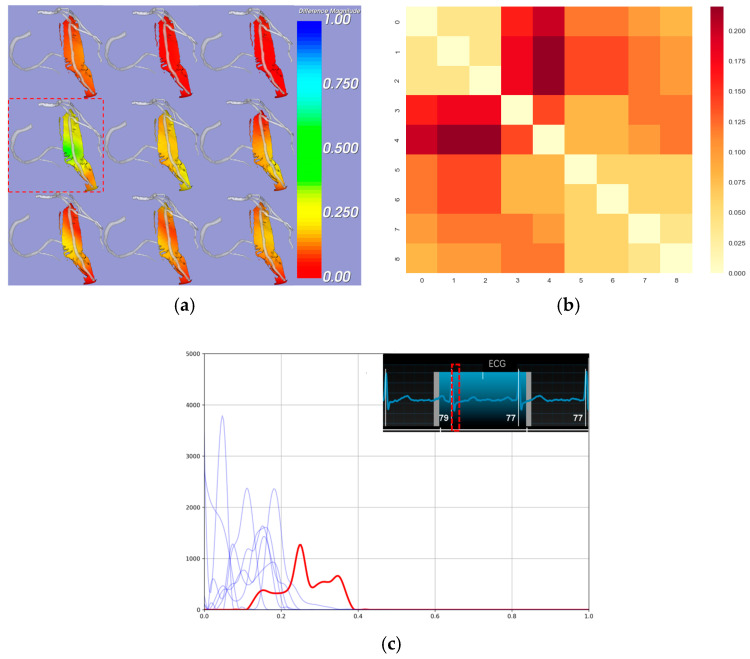
Distribution of motion disparity across cardiac phases in ROI surrounding the LAD. (**a**) Visualization of EAT–pericardium motion differences. The motion difference magnitude is color-coded on the EAT surface, with cooler colors indicating larger displacement differences between the corresponding EAT and pericardial points, representing no pericardial adhesion. The greatest relative motion difference occurs during early systole(indicated by the red dashed box). When regions appear closer to red, it indicates weak relative motion, suggesting possible pericardial adhesions. (**b**) KS statistics’ matrix of nine cardiac phases. (**c**) Histograms show the distribution of the motion difference’s magnitude at each cardiac phase. The red curve represents the phase with maximum motion disparity, which corresponds to the early systolic phase of the heart (indicated by the red dashed box area in the ECG). The blue curves show other phases. *X*-axis: normalized displacement differences (range 0.0–1.0); *Y*-axis: frequency count.

Since the pericardium exhibits no active motion, the motion difference between the pericardium and the heart is primarily driven by cardiac motion. During systole, relative motion is more pronounced due to the active contraction and rapid volume changes, while during diastole, motion is less pronounced due to the heart’s predominantly passive dilation, and the entire cardiac cycle exhibits a continuum of changes [17]. To evaluate the quality of and temporal variation in motion differences, we analyzed the distribution characteristics across cardiac phases using the two-sample Kolmogorov–Smirnov (KS) test. For each phase pair ($t_{i},t_{j}$), we computed the KS test statistic:

(10) $$D_{ij}={sup}_{x}\left|F_{i}\left(x\right)-F_{j}\left(x\right)\right|$$

where $F_{i}\left(x\right)$ and $F_{j}\left(x\right)$ represent the empirical distribution functions of motion difference magnitudes at phases i and j, respectively. This analysis yielded a KS statistics’ matrix, quantifying the dissimilarity between distributions at different phases. The KS statistics’ provide strong validation for our chosen motion difference magnitude metric. The KS matrix (range 0–0.2) reveals distinct phase-dependent patterns with appropriate diagonal consistency. The observed gradual transitions between adjacent phases and the differences between distinct phases align with expected cardiac motion patterns (Figure 3a,b). These results demonstrate that our proposed metric effectively quantifies motion disparity across cardiac phases, establishing its reliability for cardiac motion analysis.

Additionally, we observed that the most significant relative motion difference occurred in the early phases of cardiac contraction, which aligns with the principles of cardiac motion (Figure 3a,c). This finding allowed us to identify specific phases for the subsequent quantitative analysis of the ROI.

### 3.2. Quantitative Comparative Analysis of Pericardial Adhesions

**Figure 4:**
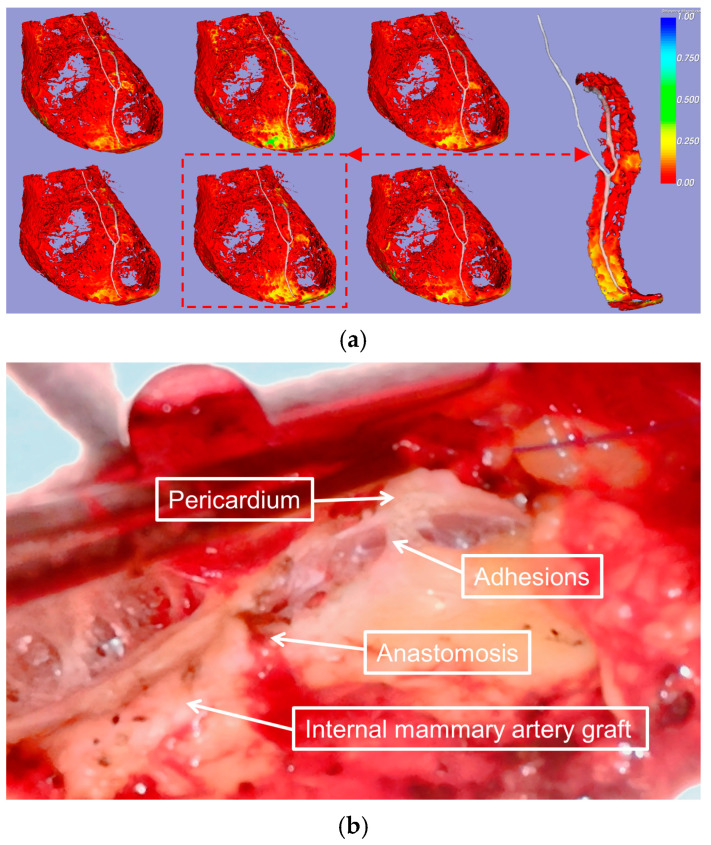
Visualization of EAT–pericardium motion differences in patients with pericardial adhesion. (**a**) The spatial distribution of EAT–pericardium motion differences was visualized across the entire epicardial fat region. The phase with maximum motion disparity (marked by a red dashed box) was selected based on the normalized displacement differences, with a magnified display focusing on the ROI surrounding the LAD for detailed examination. (**b**) This is an intraoperative photograph from a preoperative case (patient No. 1). The patient’s previous surgery, performed one year prior, consisted of a Morrow procedure, MVR, and CABG with the left internal mammary artery to the LAD. During the current operation, extensive pericardial adhesions were noted in the surgical field, particularly around the anastomotic site.

The reduced relative motion observed in cases of pericardial adhesions was consistent with the intraoperative findings (Figure 4). All raw data analyzed in this study are available in the Supplementary Material.

To evaluate the distributional characteristics of the pericoronary motion differences between the EAT and pericardium, we propose a comprehensive quantitative framework based on novel statistical metrics.

The peak ratio (PR) is defined as:

(11) $$PR=\frac{H_{max}}{H_{\alpha }}$$

where $H_{max}$ represents the maximum frequency value of the normalized motion differences, and $H_{\alpha }$ denotes the frequency value at the αth percentile bin (empirically set to the 25^th^ percentile in this study). This metric effectively captures the steepness and decay characteristics of the motion difference distribution along the LAD segments. Additionally, we introduce the distribution width index (DWI), expressed as

(12) $$DWI=\frac{N_{nonzero}}{N_{total}}$$

where $N_{nonzero}$ represents the count of nonzero frequency bins, and $N_{total}$ denotes the total number of bins within the defined cylindrical ROI around the LAD centerline.

Statistical analysis revealed distinct patterns between the adhesion and non-adhesion groups. The adhesion group demonstrated high PR values (>100) coupled with low DWI values (<0.3), indicating highly concentrated motion differences with rapid decay, suggesting restricted relative movement between the EAT and pericardium. In contrast, the non-adhesion group exhibited moderate PR values (<50) and higher DWI values (>0.4), reflecting broader distributional patterns with multiple peaks, indicating more natural tissue mobility.

**Figure 5:**
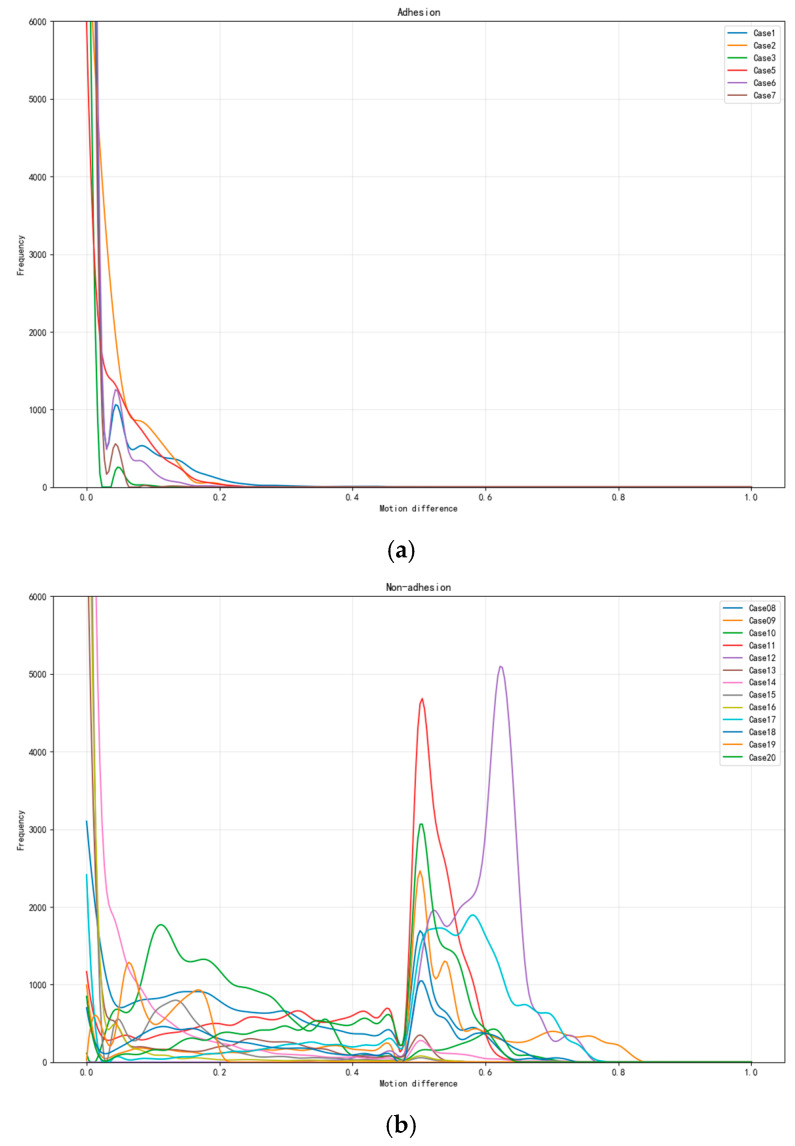
Frequency distribution analysis of adhesion and non-adhesion patterns under varying motion differences. These distribution curves present the relationship between the motion and area metrics, where the *X*-axis denotes the relative motion difference as a normalized velocity indicator, and the *Y*-axis represents the frequency distribution quantified by the cardiac surface voxel counts. The *Y*-axis frequency indicator is expressed as a normalized area metric, with each point along the curve reflecting the normalized area distribution corresponding to its specific velocity magnitude. (**a**) The frequency distribution of adhesion patterns for seven experimental cases (Case1–Case7) shows exponential decay with increasing motion difference and peak frequencies near zero motion. (**b**) Frequency distribution of non-adhesion patterns for 13 experimental cases (Case8–Case20), demonstrating distinct peaks at motion differences between 0.5 and 0.6, with Case15 exhibiting the highest frequency (~5000). Both analyses reveal contrasting behavioral patterns in adhesion and non-adhesion characteristics across different motion ranges (0–1.0).

We conducted comprehensive statistical testing to validate these metrics across the standardized ROI along the middle and distal LAD segments. The results demonstrate statistically significant differences between the groups (*p* < 0.01). The PR matrix (range 100–150 for the adhesion group, 20–50 for the non-adhesion group) shows clear group-specific patterns, while the DWI values (range 0.2–0.3 for the adhesion group, 0.4–0.6 for the non-adhesion group) further confirm the distinct motion characteristics (Figure 5).

These findings demonstrate that our proposed metrics effectively quantify pericoronary motion differences, establishing their reliability for adhesion pattern analysis. The combination of PR and DWI provides a robust framework that captures complementary aspects of tissue motion characteristics while maintaining computational efficiency and anatomical relevance.

## 4. Discussion

Studies have shown that the pericardial thickening or calcification observed on CT images alone is insufficient to rule out the presence of pericardial adhesions. Conversely, these features alone are also inadequate to confirm the diagnosis of pericardial adhesions [18]. The primary principle in assessing pericardial adhesions should be the relative motion between the pericardium and the heart, making dynamic evaluation essential. The framework proposed in this study assesses pericardial adhesions by dynamically reconstructing the heart’s pericardium and the outermost layer, the EAT. In recent years, EAT has been extensively studied due to its strong association with the development of various cardiovascular diseases, including coronary artery disease, heart failure, and atrial fibrillation [19]. CT has become a standardized imaging tool for evaluating epicardial fat, with mature and increasingly standardized methods for its segmentation and quantification [20]. Our findings affirm the reliability and robustness of both the EAT segmentation approach and the proposed motion analysis method: the KS statistics’ matrix (ranging from 0 to 0.2) revealed distinct, phase-dependent motion patterns with high consistency. The gradual changes between adjacent phases validated the reliability of the proposed motion difference metric. Meanwhile, this study demonstrated that the relative motion differences between the EAT and the pericardium were most pronounced during early systole. This insight establishes a reliable phase reference for further quantitative assessments of pericardial adhesion severity.

The multimodal imaging and diagnosis of pericardial adhesions have garnered significant interest, and ultrasound and cardiac MRI can also be dynamically observe and record cardiac activity as well as 4D CT [21]. While high-frequency ultrasound can detect pericardial thickening and its relative motion [22], it requires a suitable imaging window and has limited tissue characterization capabilities. Cardiac MRI provides a detailed depiction of pericardial thickness and its dynamic features. Some studies have defined pericardial adhesions and performed subjective evaluations by observing the sliding motion between the pericardium and the heart, but quantitative assessment methods remain lacking [7]. Unlike previous studies that often relied on two-dimensional observations, the method proposed in this study enables a comprehensive three-dimensional assessment of pericardial adhesions across different cardiac regions. Through 3D observation, the surgeon can clearly correspond the adhesions to the cardiac structures, providing more spatial location information for reference in developing the surgical plan. Cardiac kinematics encompass complex composite movements characterized by both anisotropic properties and temporal sequencing patterns. Therefore, the demonstration of pericardial adhesion patterns across all cardiac phases is of paramount importance for comprehensive evaluation. In our heatmap analysis paradigm, the presence of cooler chromatic regions during any single temporal phase indicates preserved relative motion in that specific anatomical region, suggesting the absence of pericardial adhesions. Conversely, regions exhibiting warmer chromatic patterns signify restricted motion dynamics, indicative of established pericardial adhesion formation. Furthermore, when analyzing the ROI’s distribution curve, peaks positioned toward the right of the spectrum indicate pronounced relative motion, similarly suggesting non-adhesion. This study only conducted quantitative analysis on the ROI around the LAD, but this method can be extended to cardiac regions of any size and location. By incorporating metrics such as the PR and DWI, this method provides a robust framework for identifying localized adhesions, as localized adhesions have also been reported [23].

Since we can measure the specific values of relative motion for any region of interest, we plan to collect additional cases in the future to determine the relative motion values associated with different levels of adhesion. This will allow us to establish quantitative grading indices for varying adhesion conditions, providing more accurate references for clinical decision making.

### Study Limitations

In patients with low volumes of EAT, the area available for assessment may be reduced. Additionally, in cases where pericardial labeling is not feasible—such as in patients undergoing their first surgery without pericardial closure or those with congenital pericardial defects—this method cannot be applied for evaluation.

## 5. Conclusions

This study presents a novel, noninvasive approach for quantifying pericardial adhesions using 4D CT imaging. By analyzing the relative motion between the EAT and the pericardium, we assessed pericardial adhesions using two new metrics (PR and DWI) and demonstrated early systole as the optimal phase for adhesion analysis. This method’s ability to quantify adhesion characteristics across any ROI and allow 3D observation highlights its clinical versatility. Future studies aim to expand this framework by incorporating larger datasets to define motion thresholds, thereby providing more precise guidance for clinical decision making.

## Figures and Tables

**Table 1:** Patient demographic data.

Patient No.	Age (y)	Sex	BMI	Present Operation	Previous Operation	Interval (y)
1	48	M	22	Vegetation removal	Morrow + MVR + CABG	1
2	58	F	23.4	MVR	ASDR	7
3	51	M	27.3	TVR	AVSDR	35
4	50	M	24.6	MVR	MVP	31
5	46	M	25.9	MVR	AVSDR	37
6	43	M	25.1	AVR	AVR	0.3
7	77	M	24.2	TVP	DVR	11
8	48	M	24.8	AVR	-	-
9	48	M	30.1	CABG	-	-
10	54	M	29.1	CABG	-	-
11	39	M	22.7	MVR + CABG	-	
12	48	M	28.1	CABG	-	-
13	43	M	29.5	MVR	-	-
14	57	M	24.4	AVR	-	-
15	39	M	25.8	CABG	-	-
16	61	M	29.5	CABG	-	-
17	55	M	28	CABG	-	-
18	65	F	30.8	CABG	-	-
19	45	M	27.7	CABG	-	-
20	62	M	26.6	CABG	-	-

BMI, body mass index; Morrow, Morrow procedure; MVR, mitral valve replacement; MVP, mitral valve plasty; CABG, coronary artery bypass grafting; ASD, atrial septal defect; AVSDR, atrioventricular septal defect repair; TVR, tricuspid valve replacement; TVP, tricuspid valve plasty; AVR, aortic valve replacement; DVR, double valve replacement (mitral valve + aortic valve).

## Data Availability

The original contributions presented in this study are included in this article/Supplementary Material. Further inquiries can be directed to the corresponding author(s).

## Supplementary Materials

The following supporting information can be downloaded at: https://www.mdpi.com/article/10.3390/bioengineering12030224/s1. Raw processing data for all patients are included in the Supplementary Material.

## Abbreviations

The following abbreviations are used in this manuscript:

CT	Computed tomography
CTA	Computed tomography angiography
4D CT	Four-dimensional computed tomography
EAT	Epicardial adipose tissue
CMR	Magnetic resonance imaging
ROI	Region of interest
CABG	Coronary artery bypass grafting
LAD	Left anterior descending
LCX	Left circumflex
RCA	Right coronary artery
KS test	Kolmogorov–Smirnov test
PR	Peak ratio
DWI	Distribution width index
